# Regulation of Peroxisome Proliferator-Activated Receptor Pathway During Torpor in the Garden Dormouse, *Eliomys quercinus*

**DOI:** 10.3389/fphys.2020.615025

**Published:** 2020-12-21

**Authors:** Alexander J. Watts, Samantha M. Logan, Anna Kübber-Heiss, Annika Posautz, Gabrielle Stalder, Johanna Painer, Kristina Gasch, Sylvain Giroud, Kenneth B. Storey

**Affiliations:** ^1^Department of Biology, Carleton University, Ottawa, ON, Canada; ^2^Department of Interdisciplinary Life Sciences, Research Institute of Wildlife Ecology, University of Veterinary Medicine Vienna, Vienna, Austria

**Keywords:** PPAR, PGC-1 alpha, hibernation, fatty acids, adipose, liver

## Abstract

Differential levels of n-6 and n-3 essential polyunsaturated fatty acids (PUFAs) are incorporated into the hibernator’s diet in the fall season preceding prolonged, multi-days bouts of torpor, known as hibernation. Peroxisome proliferator-activated receptor (PPAR) transcriptional activators bind lipids and regulate genes involved in fatty acid transport, beta-oxidation, ketogenesis, and insulin sensitivity; essential processes for survival during torpor. Thus, the DNA-binding activity of PPARα, PPARδ, PPARγ, as well as the levels of PPARγ coactivator 1α (PGC-1α) and L-fatty acid binding protein (L-FABP) were investigated in the hibernating garden dormouse (*Eliomys quercinus*). We found that dormice were hibernating in a similar way regardless of the n-6/n-3 PUFA diets fed to the animals during the fattening phase prior to hibernation. Further, metabolic rates and body mass loss during hibernation did not differ between dietary groups, despite marked differences in fatty acid profiles observed in white adipose tissue prior and at mid-hibernation. Overall, maintenance of PPAR DNA-binding activity was observed during torpor, and across three n-6/n-3 ratios, suggesting alternate mechanisms for the prioritization of lipid catabolism during torpor. Additionally, while no change was seen in L-FABP, significantly altered levels of PGC-1α were observed within the white adipose tissue and likely contributes to enhanced lipid metabolism when the diet favors n-6 PUFAs, i.e., high n-6/n-3 ratio, in both the torpid and euthermic state. Altogether, the maintenance of lipid metabolism during torpor makes it likely that consistent activity or levels of the investigated proteins are in aid of this metabolic profile.

## Introduction

Before the onset of predictable resource scarcity during winter, small mammals such as the garden dormouse (GD, *Eliomys quercinus*), prepare for entering a period of several bouts of multi-days torpor, i.e., hibernation, by engaging in hyperphagia and reducing their metabolic rate in the preceding fall season (Sheriff et al., 2012, 2013). Unique adaptations have evolved to allow hibernators to suppress their global rate of metabolism while catabolizing mainly fatty acids and keeping carbohydrate stores reserved (Dark, 2005; Wu et al., 2013), in an effort to sustain themselves until spring; this is in contrast to food-storing mammals which survive on food caches (Weitten et al., 2016). By increasing their ingestion of fat-laden foods and altering circulating hormone levels, fat-storing mammalian hibernators increase their weight by around 40% usually before lowering activity levels and body temperature (T_*b*_) in the late summer and early fall (Pengelley and Fisher, 1966; Mrosovsky, 1977; Geiser, 2016). During the hibernation season enhanced fat stores are utilized as metabolic fuels allowing them to survive the entire winter season. Notably, the enrichment of certain types of lipids, namely unsaturated fatty acids and especially n-6 polyunsaturated fatty acids (PUFA) are more beneficial for heterotherms than others and confer the ability to survive winter (see for reviews: Munro and Thomas, 2004; Dark, 2005; Ruf and Arnold, 2008; also from a large hibernator: Giroud et al., 2019); within adipocytes, due to the increased reliance on beta-oxidation for energy production, as well as in other tissues for the maintenance of membrane fluidity and proper protein functioning at low temperatures (for review, see Arnold et al., 2015; Staples, 2016; Giroud et al., 2018).

The ratio of certain fats within a mammalian hibernator’s diet becomes consequential during their sustained dependence on lipid metabolism since these lipids also become incorporated and enriched in the cellular membranes of tissues that affect lipid catabolism (Geiser et al., 1994; Dark, 2005). Hibernators that consume a diet enriched with plant oils, which tend to be high in PUFAs, have lengthened torpor bouts and decreased torpid T_*b*_, which could preserve energy for extended heterothermy (Geiser and Kenagy, 1987; Frank, 1992; Florant et al., 1993; Munro et al., 2005). Importantly, n-3 PUFAs (e.g., 18:3 n-3, i.e., α-linolenic acid, ALA) tend to effect hibernation in ways opposite to n-6 fatty acids (e.g., 18:2 n-6, i.e., linoleic acid, LA) PUFAs (Ruf and Arnold, 2008; Arnold et al., 2015). Hibernating species that are fed a diet high in n-6 but low in n-3 fatty acids are more likely to enter and remain in torpor than animals fed an equivalent amount of PUFAs but with a reversed ratio (low n-6/n-3) (Hill and Florant, 2000; Frank et al., 2008). Clearly then, the makeup of essential fatty acids consumed during the hibernator’s preparatory period impacts the overall success of hibernation as a survival-strategy.

Indeed, the composition of phospholipid membranes can affect a number of intracellular pathways. One impact of lowered membrane n-3 levels is altered expression of the genes downstream of 2 transcription factor groups: the sterol-regulatory-element binding proteins (SREBPs) and peroxisome proliferator-activated receptors (PPARs) (for review see: Deckelbaum et al., 2006). Interestingly, these transcription factors are deeply involved in transcribing fatty-acid metabolism genes, as well as genes important in other processes such as inflammatory responses (Wahli and Michalik, 2012; Shimano and Sato, 2017). Given their influence over fatty acid metabolism, the regulation of PPARs has been explored in other hibernating species, including the little brown bat (*Myotis lucifugus*) and the 13-lined ground squirrel (*Ictidomys tridecemlineatus*) (Eddy and Storey, 2003; Eddy et al., 2005), wherein findings showed that PPARγ was upregulated during torpor. Upon binding a fatty acid ligand, PPARs bind a co-activator protein that allows transcription of genes containing a PPAR-response element. For instance, the cold-inducible PPARγ coactivator-1α (PGC-1α) regulates mitochondrial metabolism linking PPARα to the thermogenic capacity of tissues in a mechanism shown to be relevant in liver from hibernating jerboas (El Kebbaj et al., 2009). PPAR also regulates lipid and energy metabolism by inducing the expression of downstream genes such as fatty-acid binding protein (FABP), a key protein involved in facilitating lipid mobilization. Finally, PPAR protein and downstream gene expression is also relevant to the recruitment and activation of beige-like cells in white adipose tissue (WAT) (Chayama et al., 2018). In summary, PPARs are intricately involved in a variety of essential processes owing to the importance of this regulatory network in the context of mammalian hibernation.

When hibernators are in a hypometabolic state, gene expression for countless cellular pathways must be intricately regulated to prevent cell stress. A greater understanding of fatty acid metabolism during hibernation, when most tissues select lipids over carbohydrates as the preferred fuel source, has provided new perspectives into the survival of cells during hypometabolism and the treatment of metabolic disorders including insulin sensitivity (i.e., diabetes) and obesity (Wu et al., 2013; Logan et al., 2016). However, few studies to date have assessed the impact of diets containing different ratios of n-6 and n-3 PUFAs on fatty acid metabolism during torpor. Herein, liver, brown adipose tissue (BAT) and WAT from GD fed either a low, intermediate, or high n-6/n-3 ratio diet, were used to assess the impact of diet in the regulation of fatty acid metabolism during hibernation. Specifically, DNA binding activity of PPARs (PPARα, δ, and γ) were assessed in conjunction with total protein levels of the cofactor PGC1-α and downstream effector protein, liver-FABP (L-FABP). Altering the lipid composition of the GD’s pre-hibernation diet has been shown to change the molecular phenotype displayed during hibernation in mammals (Logan et al., 2020), making it likely that molecular changes in fatty acid metabolism pathways will result from this difference.

## Methods

### Animals

In total, 41 garden dormice (*Eliomys quercinus*) weighing prior to hibernation 140.0 ± 2.6 g [116-170 *g* CI] obtained from a breeding colony kept at the Research Institute of Wildlife Ecology (Vienna, Austria) were included in these experiments (Table 1). Animals were housed singly in cages (60 × 40 × 40 cm), each equipped with one nest, bedding and nesting material. Dormice were kept under natural fluctuations of ambient temperature (T_*a*_) and photoperiod during their pre-hibernation fattening (September), until the hibernation period (October to January) under constant darkness, without food and water. During this time dormice were housed individually in standard laboratory cages (36 × 20 × 14 cm), each provided with a customized nest and bedding material, and kept at 4°C in ventilated cooling units (Liebherr GKv 5730).

**TABLE 1:** Sample sizes of the different measurements and analyses conducted during the hibernation experiments.

Categories	Hibernation			Mid-hibernation		Dietary groups
	Metabolic rates	T_*b*_ patterns	Lipid profiles	Molecular assessments		
Group sample sizes	*n* = 24	*n* = 32		*n* = 16		*n* = 8 (4) Low *n* = 8 (7) Inter *n* = 8 (5) High
				–		*n* = 2 Low *n* = 4 Inter *n* = 2 High
	–					
		–		*n* = 9 with T_*b*_ only at mid-hibernation		*n* = 3 Low *n* = 1 Inter *n* = 5 High
Total analyses sample sizes	*n* = 24	*n* = 32		*n* = 41	*n* = 25	*n* = 13 (7) Low *n* = 13 (8) Inter *n* = 15 (10) High

*Specific sample sizes are also indicated for each of the dietary groups, i.e., low n-6/n-3 (‘Low’), intermediate n-6/n-3 (‘Inter’), and high n-6/n-3 (‘High’) fatty acid diets. Numbers given in parentheses indicate sample sizes of the molecular analyses for each dietary group when different from the group sample sizes. Lipid profiles were assessed prior to hibernation and at mid-hibernation (sacrifices). In total, 41 garden dormice were included in the overall experiments.*

### Ethics Statement

All procedures were approved by the institutional ethics committee and the national Austrian authority in accordance to the Austrian Animal Experimentation Act, ‘Tierversuchsgesetz 2012’ (BMBWF-68.205/0137-WF/V/3b/2014).

### Protocol Overview

During the pre-hibernation fattening period, the 41 dormice were fed one of the three specific lipid diets (see below for further details), and then implanted with T_*b*_ transmitters prior to hibernation (see below for further details). After recovery from surgeries and once animals had spontaneously expressed torpor, hibernation was induced by housing the animals at 4°C without food and water. The readiness of dormice to enter prolonged torpor or hibernation was evaluated via measurements of the body mass and food intake of the individuals prior to hibernation. Once dormice attained a plateau for body mass and food intake was largely reduced, we considered that the individuals were extensively using torpor likely entering multiday torpor bouts, as previously shown in juvenile garden dormice prior to hibernation (Giroud et al., 2012, 2014; Mahlert et al., 2018). During the 3 months of experiments, core T_*b*_ via temperature transmitters was measured continuously in 32 dormice and metabolic rate (MR) using respirometry was recorded in 24 animals out of the 41 studied individuals, from which fatty acid compositions of WAT were determined at both pre- and mid-hibernation (see Table 1 for details). Nine additional animals that were also implanted with T_*b*_ transmitters, were recorded for core T_*b*_ during at least two bouts before sacrifices at mid-hibernation. Molecular data were assessed in a subset (*n* = 16) of the 32 individuals continuously followed for T_*b*_ during hibernation, and from the extra-animals (*n* = 9) from which T_*b*_ was recorded shortly before sacrifices, leading to a total of 25 dormice investigated for molecular aspects (see Table 1 for details). Animals were sacrificed at mid-winter (December to January), when torpor bout lengths are maximal, either in torpor or during interbout euthermia, by immediate decapitation (if torpid; T_*b*_: 4.55 ± 0.15°C measured via implanted transmitters) or by CO_2_-euthanasia then decapitation (if euthermic, T_*b*_: 37.05 ± 0.13°C measured via implanted transmitters). Tissues were quickly sampled and immediately flash frozen in liquid nitrogen (−196°C) and stored at −80°C for 4-12 months until shipped to Carleton University on dry ice.

### Diets

The fatty acid composition of the diets is summarized in Logan et al. (2020). During the fattening phase, dormice were fed one of the three specific diets, each differing in its lipid composition, made by adding either a 10% w/w of linseed oil as the source of n-3 fatty acids (notably ALA 18:3 n-3), or a 10% w/w of safflower oil as the source of n-6 fatty acids (mainly LA 18:2 n-6), or adding a 5% w/w of linseed oil and a 5% w/w of safflower oil to pellets the animals were accustomed to (Topix, Saturn Petcare GmbH, Bremen, Germany). Details of the exact composition of the colony diet is available in Mahlert et al. (2018). These preparations produced a chow with either low, intermediate or high amounts of LA or n-6 PUFAs, (described as such) but reversely mirrored by the contents of ALA or n-3 PUFAs (see Table 2 for summary). Fresh pellets (kept in sealed bags filled with nitrogen at −80°C) were fed to the animals every 2 days, and uneaten food was discarded. During the September fattening phase, each group of dormice was fed their respective experimental diet for at least 14 days, which was previously shown to be sufficient to ensure maximum changes in the fatty acid composition of membranes and tissues in small rodents (Swanson and Kinsella, 1986; Swanson et al., 1987). Access to the specific lipid diets was maintained until dormice were moved to the hibernating cooling units where both food and water were entirely and permanently removed during the entire hibernation experiments.

**TABLE 2:** Proportions of Linoleic acid (LA) and Linolenic acid (ALA) of linseed oil- (‘Low LA’ or ‘High ALA’), safflower oil- (‘High LA’ or ‘Low ALA’) and linseed/safflower oil (‘Inter LA’ or ‘Inter ALA’) -enriched diets as fed to garden dormice for at least two weeks prior to hibernation.

FATTY ACIDS	DIETS		
	Low LA or High ALA	Inter LA or Inter ALA	High LA or Low ALA
Linoleic acid (18:2 n-6)	19.28	35.55	52.95
Linolenic acid (18:3 n-3)	31.92	17.16	1.35
n-6 PUFA	19.62	35.87	53.29
n-3 PUFA	32.20	17.47	1.71
n-6/n-3	0.61	2.06	31.50
LA/ALA	0.61	2.08	41.22

*The sums of n-6 or n-3 PUFA, as well as the ratios between n-6 and n-3 PUFA or LA and ALA of the three specific diets are also indicated. Fatty acid proportions are expressed as% of total fatty acids. Values are means from four analyses per diet. Data were extracted from Table 1 in Logan et al. (2020).*

### Surgical Implantations of Transmitters and Body Temperature Measurements

Prior to hibernation experiments, the animals were implanted with small temperature transmitters and core T_*b*_ was monitored via a telemetry system. TA-F10 transmitters (1.1cc, 1.6g, accuracy: 0.15°C; Data Sciences International, St Paul, United States) were calibrated prior to implantation between 0 and 40°C in a temperature-controlled water bath. Surgery proceeded as previously described (Giroud et al., 2018). In short, transmitters were surgically implanted under anesthesia induced by subcutaneous 50 mg kg^–1^ ketamine (Ketamidor^®^ 10%, Richter Pharma, Wels, Austria) and 5 mg kg^–1^ xylazine (Rompun^®^ 2%, Bayer, Leverkusen, Germany) injection, which was maintained using 1.5% isoflurane via facemask. A subcutaneous administration of 5 mg kg^–1^ ketoprofen (Romefen^®^ 10%, Merial S.A.S., Toulouse, France) was provided as post-operative analgesic. Upon surgical implantation of the temperature transmitters, a small amount (10-30 mg) of subcutaneous WAT were collected from each animal. WAT-samples were flash-frozen in liquid nitrogen and stored at −80°C until subsequent analysis of fatty acid composition. Following surgery, animals recovered for ten days before starting temperature recordings. An RPC-1 receiver board (Data Sciences International) was positioned under each individual cage to collect transmitter data. A 10s T_*b*_ measurement was recorded every 5 min, and data was analyzed using the Dataquest software (LabPro Data Sciences). Several parameters were derived from the temperature recordings. We assessed the onset of hibernation as the time between the food removal in cooling units at 4°C and entrance into the first torpor bout with a T_*b*_ threshold of 18°C and lasting at least for 24h. We further determined the number of arousals, mean and total arousal and torpor durations (with a T_*b*_ threshold of 18°C), as well as minimal T_*b*_ during torpor.

### Metabolic Rate Measurement

Metabolic rate or oxygen consumption (VO_2_ in ml O_2_ h^–1^) was assessed using an open-flow respirometry system, as previously described in Nowack et al. (2019) with the following modifications. We used a dual-channel electrochemical oxygen analyzer (FC-2 differential oxygen analyzer Oxzilla, Sable System, Las Vegas, United States^1^), which was calibrated once prior to the experiments with nitrogen for zero-oxygen value. During the hibernation experiments, we used the auto-calibration function of Oxzilla to reset the oxygen concentration to reference air at regular intervals. Air was pumped through the respirometry chamber (pulled mode) by mean of membrane pumps with a flow rate of ∼40 L h^–1^. Relative humidity was measured in sampled air and used for correction within the calculations. VO_2_ was measured in a group of 24 dormice (eight from each dietary group), where six individuals were measured in parallel. Oxygen measurements were corrected for drift of the analyzer by automated switching to reference air at regular intervals. MR was computed using a self-written R-program including the following equation VO_2_ (L O_2_ h^–1^) = FD^∗^(FIO_2_-FEO_2_)/(1-FIO_2_^∗^(1-RQ)), where FD = dry flow, FIO_2_ = fraction O_2_ concentration in the incoming airflow, FEO_2_ = fraction O_2_ concentration in the outgoing airflow, RQ = respiratory quotient, by Lighton (2008), assuming a RQ of 0.7. From VO_2_ measurements, we visually determined interbout euthermic phases and computed the averaged MR during interbout euthermia (‘mean euthermic MR’). Then, we defined a VO_2_ threshold for torpor bouts as 25% of the mean euthermic MR and computed the averaged MR during torpor (‘mean torpid MR’) which includes VO_2_ values below the threshold. We further calculated the averaged MR during the entire hibernation experiments, i.e., from multiday torpor-arousal cycles (‘mean hibernating MR’) over the experiments.

### Lipid Analysis

Total lipids were extracted from WAT at both pre-hibernation (i.e., during surgeries) and mid-hibernation following the procedure of Folch et al. (1957). Since triglyceride fatty acids represent more than 95% of total lipids in rodent WAT (Florant et al., 1990), triglycerides and phospholipids in WAT were not separated prior to analysis. Samples were trans-esterified with a one-step method (Lepage and Roy, 1986; Eder, 1995). As previously described by Giroud et al. (2018), Fatty acid methyl esters (FAME) were identified by gas-liquid chromatography using a FID AutoSystem XL autosampler chromatograph (Perkin-Elmer, Traiskirchen, Austria) equipped with a 30 m × 0.25 mm × 0.25 μm HP INNOWax capillary column, using the following parameters: injector 240°C, column 130–180°C at 4°C/min, 180–200°C at 3°C/min, 200–240°C at 15°C/min, 240°C for 8 min. The relative fatty acid composition was quantified using Supelco external FAME standards (Sigma-Aldrich Handels GmbH, Vienna, Austria) run after every 20 samples and Turbochrom 6.3 software (Perkin Elmer). The concentrations of single fatty acids were calculated as mass% of total identified peaks for 13 fatty acids that had a chain length of between 14 and 22. We further computed the sums of PUFAs, monounsaturated fatty acids (MUFA), saturated fatty acids (SFA), n-6 or n-3 PUFAs, as well as the ratio between n-6 and n-3 PUFA (n-6/n-3).

### Total Protein Extraction

Total soluble protein was extracted from frozen liver, WAT and BAT. Frozen tissue was weighed (∼50-75 mg) and homogenized using a dounce-homogenizer in ice-cold cell lysis buffer (EMD Millipore, Billerica, MA; catalog No. 43-040) with added phosphatase (1 mM Na_3_VO_4_, 10 mM ß-glycerophosphate and 10 mM NaF) and protease (BioShop; catalog No. PIC001) inhibitors, using ratios of 1:5 (w/v) for liver and BAT and 1:3 (w/v) for WAT. Cell lysis was allowed to proceed on ice for 30 min with occasional agitation, before centrifuging for 20 min at 4°C and 12 000 × *g.* Total soluble protein was collected as the supernatant. A Bradford assay (Bio-Rad; catalog No. 500-0005) was employed to determine protein concentrations from tissue extracts and to standardize samples to 10 μg/μl before storage at −80°C.

### Analysis of PPAR DNA-Binding Activity

The activity of several PPAR targets relevant to fatty acid metabolism were investigated in total protein extracts from aroused (control) and torpid garden dormouse. Measurements of DNA binding activity were conducted for PPARα, PPARδ, PPARγ (Abcam, Cambridge, MA, United States; catalog Nos. ab133107, ab133106, and ab133101, respectively), as previously published (Sharif et al., 2014; Ghosh et al., 2019; Jiang et al., 2020). DNA binding activity was measured according to the manufacturer’s protocol. Following quality control and validation studies (i.e., determination of optimal protein load), consistent amounts of extracted protein (100 μg per well) were combined with the provided transcription factor binding buffer. Standardized total protein extracts were applied to microplate containing a specific double-stranded DNA probe containing the peroxisome proliferator response element (PPRE) immobilized in the bottom of the wells. Samples were incubated overnight at 4°C and provided primary and secondary antibodies were added following washes with the supplied wash buffer. Absorbance in each well was read at 450 nm using a Powerwave HT spectrophotometer (BioTek; Winooski, VT, United States). Wells with additional transcription factor binding buffer instead of protein extracts were run alongside the assay to act as negative controls as per the manufacturer’s instructions.

### Analysis of FABP and PGC-1 Levels

Protein levels of FABP and PGC-1α were also investigated in both aroused (control) and torpid conditions. FABP measurements were conducted in liver and made use of the Mouse Liver-FABP ELISA Kit (Abcam; catalog No. ab218262). For FABP measurements, 0.25 μg of protein per well was incubated with an antibody cocktail containing both primary and secondary antibodies for 1 h. Following the addition of TMB substrate for 10 min, the absorbance in each well was then read at 450 nm using a Powerwave HT spectrophotometer (BioTek, Winooski, VT, United States).

PGC-1 measurements were conducted using 2.5 μg of protein per well; incubated for 2 h in pre-coated ELISA wells using the mouse PPARGC1A ELISA kit (MyBioSource, San Diego, CA, United States; catalog No. MBS707053). Manufacturer-provided primary and secondary antibodies were incubated for 1 h each following washes and well absorbance was detected at 450 nm using a Powerwave HT spectrophotometer (BioTek) following 7 min of color development with TMB. Wavelength correction was applied using additional readings at 540 nm and 570 nm. For both ELISA assays, wells with additional sample diluent buffer instead of protein extracts were assayed as negative controls as per the manufacturer’s instructions.

### Statistical Analysis

We used R 3.5.1 (R Core Team, 2018) to perform statistical analyses of fatty acid composition, hibernating patterns, MRs, body mass loss over the hibernation experiments. The distribution of statistical model residuals was assessed by inspecting quantile-quantile-plots and histograms. When necessary, response variables were Box-Cox transformed to achieve normality. We used linear mixed-effects models (R package ‘nlme’) (Pinheiro et al., 2014) with animal ID as random factor to test effects of dietary treatment (low, intermediate and high LA) and time (pre-hibernation vs. mid-hibernation) on WAT fatty acid composition. We reused previously published data of WAT fatty acid composition at pre-hibernation from Logan et al. (2020) into the analyses to compare changes in fatty acid composition during the experimental hibernation period. All *p*-values from linear mixed-effects models were adjusted for multi-comparisons between fatty acid proportions using False Discovery Rate (Benjamini and Hochberg, 1995). Tukey-like *post hoc* multiple comparison tests (R package ‘multicomp’) (Hothorn et al., 2008) were applied to test for specific differences between dietary groups and periods. Dietary effects on body mass loss over hibernation was also tested by using a linear mixed-effects model with post-hibernation bod mass as a response variable and pre-hibernation body mass along with diet treatment as fixed factors. We further employed linear mixed-effects models with pre-hibernation body mass as a random factor to test effects of diets on variables derived from the hibernating patterns and MR measurements. Because dormice were sacrificed within a two-week period, individual duration of experimental hibernation was included as a random factor in the model for the number of arousals as well as total arousal and torpor durations.

Fold change values of molecular variables were calculated by comparing the difference between a particular diet (low, intermediate or high) to the euthermic intermediate value which is normalized to 1.0, or by comparing differences in the state (aroused or torpid) to the same. For example, the fold change for the low diet value was compared to the intermediate diet value, expressed as low/intermediate, and where a fold change > 1 represents an increase, while a fold decrease is expressed as the percentage decrease and represents a fold change < 1. All numerical data are expressed as mean with data points (*n* = 4 samples from independent animals) and were graphed using ggplot2 in R (v. 3.6.1) (Wickham, 2016). Statistical analysis of differences between experimental hibernation time-points was performed using a one-way ANOVA and *post hoc* Tukey tests using RBioPlot statistical package (Zhang and Storey, 2016) with *p* < 0.05 accepted as significant. All reported values are means ± SE.

## Results

### WAT Lipid Profiles Prior to Hibernation and at Mid-Hibernation

After at least 14 days of feeding specific diets contrasting in their n-6 to n-3 PUFA ratio during the fattening phase, the pre-hibernation WAT fatty acid composition of dormice substantially differed between dietary groups (Tables 3, 4). Specifically, proportions of 18:2 n-6 (LA) and 18:3 n-3 (ALA) were significantly higher and lower, respectively, in WAT of dormice fed diets with increased n-6/n-3 PUFA (Table 3). These differences led to contrasted sums of n-6 or n-3 PUFA and n-6/n-3 ratios between the three dietary groups. We further found lower proportions of 20:4 n-6 and higher levels of 18:1 n-9 in WAT of dormice fed a low n-6/n-3 diet compared to high n-6/n-3 diet-fed individuals, while proportions of those fatty acids in WAT of intermediate n-6/n-3 diet-fed individuals did not differ from the other two groups (Table 3). Also, long-chain n-3 fatty acids, namely 20:5 n-3, 22:5 n-3, and 22:6 n-3, were significantly in higher proportions in WAT of dormice fed a low or an intermediate n-6/n-3 diet compared to those from individuals fed a high n-6/n-3 diet. Finally, WAT levels of all SFAs as well as the sum of SFA were similar across all dietary groups.

**TABLE 3:** Parameters of linear mixed-effects models with animal ID as random factor for the effects of diet treatment (‘Diet’), time (‘Time,’ pre-hibernation vs. mid-hibernation), and their interaction (‘Diet*Time’) on fatty acid proportions (% of total fatty acids) and ratios of certain fatty acid proportions from white adipose tissue total lipids of garden dormice fed one of the three diets contrasting in their n-6/n-3 PUFA ratio.

Fatty acids	Diet		Time		Diet*Time	
	*F*-Value	*p*-Value	*F*-Value	*p*-Value	*F*-Value	*p*-Value
C14:0	0.70	0.54	0.82	0.44	0.66	0.66
C15:0	3.14	0.07	0.75	0.44	2.64	0.24
C16:0	2.18	0.16	124.14	<*0.001*	0.18	0.89
C16:1 n-7	4.71	*0.026*	19.62	<*0.001*	1.23	0.48
C17:0	1.65	0.25	1.39	0.32	1.55	0.42
C18:0	0.04	0.96	32.63	<*0.001*	4.59	*0.06*
C18:1 n-9	17.21	<*0.001*	0.51	0.51	9.49	<*0.001*
C18:2 n-6	250.91	<*0.001*	72.60	<*0.001*	1.13	0.50
C18:3 n-3	797.18	<*0.001*	10.66	*0.003*	0.73	0.66
C20:4 n-6	9.85	*0.008*	17.97	<*0.001*	1.70	0.42
C20:5 n-3	5.43	*0.015*	20.07	<*0.001*	9.24	<*0.001*
C22:5 n-3	4.48	*0.029*	7.10	*0.015*	4.30	0.07
C22:6 n-3	4.36	*0.029*	17.35	<*0.001*	2.17	0.31
PUFA	17.05	<*0.001*	27.27	<*0.001*	10.34	<*0.001*
MUFA	16.32	<*0.001*	0.28	0.60	8.96	<*0.001*
SFA	1.34	0.30	51.11	<*0.001*	0.12	0.89
n-6	253.22	<*0.001*	69.14	<*0.001*	1.27	0.49
n-3	692.41	<*0.001*	11.87	*0.002*	0.55	0.69
n-6/n-3	699.08	<*0.001*	32.60	<*0.001*	0.22	0.89

*‘PUFA’ refers to polyunsaturated fatty acids, ‘MUFA’ to monounsaturated fatty acids, ‘SFA’ to saturated fatty acids, ‘n-6’ to the sum of n-6 PUFA, ‘n-3’ to the sum of n-3 PUFA, and ‘n-6/n-3’ to the ratio between the sum of n-6 PUFA and the sum of n-3 PUFA. Significant p-Values are shown in italic.*

**TABLE 4:** Fatty acid proportions (% of total fatty acids), prior to and at mid-hibernation of white adipose tissue total lipids (means ± standard error), and ratios of certain fatty acid proportions of garden dormice fed diets of either low (‘LOW’), intermediate (‘INTER’) or high n-6/n-3 PUFA ratio (‘HIGH’).

Fatty acid	Pre-hibernation			Mid-hibernation		
	LOW	INTER	HIGH	LOW	INTER	HIGH
C14:0	1.58 ± 0.14	1.63 ± 0.11	1.63 ± 0.07	1.58 ± 0.08	1.63 ± 0.05	1.59 ± 0.05
C15:0	0.03 ± 0.01	0.02 ± 0.01	0.01 ± 0.01	0.08 ± 0.05	0.06 ± 0.02	0.01 ± 0.01
C16:0	12.31 ± 0.82	12.96 ± 0.45	12.80 ± 0.37	9.89 ± 0.58^*^	10.65 ± 0.29^*^	10.66 ± 0.22^*^
C16:1 n-7	4.84 ± 0.12^*a*^	5.26 ± 0.21^*a*^	4.65 ± 0.23^*a*^	4.56 ± 0.16^*ab*^	4.65 ± 0.20^*a*^	3.95 ± 0.16^*b*^
C17:0	0.05 ± 0.02	0.06 ± 0.02	0.03 ± 0.01	0.14 ± 0.06	0.06 ± 0.02	0.03 ± 0.02
C18:0	2.15 ± 0.08^*a*^	2.09 ± 0.07^*a*^	1.95 ± 0.08^*a*^	2.27 ± 0.08^*a*^	2.28 ± 0.08^*a*^	2.40 ± 0.11^a*^
C18:1 n-9	46.57 ± 0.96^*a*^	45.48 ± 0.70^*ab*^	42.82 ± 1.05^*b*^	49.46 ± 0.77^*a*^	45.03 ± 0.44^*b*^	41.68 ± 0.65^*b*^
C18:2 n-6	14.04 ± 0.73^*a*^	22.85 ± 0.69^*b*^	34.76 ± 1.12^*c*^	15.51 ± 0.48^*a*^	26.71 ± 0.59^b*^	39.79 ± 0.79^*c*^
C18:3 n-3	17.85 ± 1.07^*a*^	9.09 ± 0.56^*b*^	0.91 ± 0.17^*c*^	16.18 ± 0.51^*a*^	8.54 ± 0.36^*b*^	0.56 ± 0.04^*c*^
C20:4 n-6	0.27 ± 0.02^*a*^	0.29 ± 0.01^*ab*^	0.38 ± 0.02^*b*^	0.19 ± 0.02^*a*^	0.26 ± 0.03^*ab*^	0.28 ± 0.02^b*^
C20:5 n-3	0.13 ± 0.04^*a*^	0.09 ± 0.02^*a*^	0.01 ± 0.01^*b*^	0.04 ± 0.01^a*^	0.04 ± 0.01^*a*^	0.02 ± 0.01^*a*^
C22:5 n-3	0.07 ± 0.02^*a*^	0.07 ± 0.02^*a*^	0.01 ± 0.01^*b*^	0.04 ± 0.01^*a*^	0.05 ± 0.01^*a*^	0.02 ± 0.01^*a*^
C22:6 n-3	0.09 ± 0.03^*ab*^	0.11 ± 0.03^*a*^	0.03 ± 0.01^*b*^	0.04 ± 0.02^*a*^	0.04 ± 0.02^a*^	0.01 ± 0.01^*a*^
PUFA	32.47 ± 0.82^*a*^	32.51 ± 1.05^*a*^	36.11 ± 1.05^*b*^	32.54 ± 0.54^*a*^	36.05 ± 0.79^*b*^	40.33 ± 0.83^c*^
MUFA	51.41 ± 1.02^*a*^	50.74 ± 0.87^*b*^	47.47 ± 1.22^*b*^	54.02 ± 0.67^*a*^	49.68 ± 0.58^*a*^	45.63 ± 0.76^*b*^
SFA	16.12 ± 0.98	16.76 ± 0.51	16.42 ± 0.47	13.96 ± 0.62^*^	14.68 ± 0.30^*^	14.69 ± 0.33^*^
Σ n-6	14.31 ± 0.74^*a*^	23.14 ± 0.68^*b*^	35.14 ± 1.13^*c*^	15.70 ± 0.47^*a*^	26.97 ± 0.59^b*^	39.07 ± 0.79^c*^
Σ n-3	18.14 ± 1.12^*a*^	9.36 ± 0.62^*b*^	0.96 ± 0.17^*c*^	16.30 ± 0.51^*a*^	8.67 ± 0.39^*b*^	0.61 ± 0.05^*c*^
n-6/n-3	0.85 ± 0.10^*a*^	2.60 ± 0.18^*b*^	46.78 ± 3.45^*c*^	0.98 ± 0.06^*a*^	3.19 ± 0.17^b*^	70.21 ± 5.37^c*^

*‘PUFA’ refers to polyunsaturated fatty acids, ‘MUFA’ to monounsaturated fatty acids, ‘SFA’ to saturated fatty acids, ‘n-6’ to the sum of n-6 PUFA, ‘n-3’ to the sum of n-3 PUFA, and ‘n-6/n-3’ to the ratio between the sum of n-6 PUFA and the sum of n-3 PUFA. Groups differing significantly with p < 0.05 (Tukey-like post hoc comparisons) are denoted by different superscript letters for differences between diets and by stars for differences between times (pre- vs. mid-hibernation) when it applies.*

During hibernation and at mid-hibernation, both LA and ALA proportions in WAT were still differing significantly between the three dietary groups, with higher levels of LA and lower proportions of ALA in WAT of dormice fed a high n-6/n-3 diet, levels which were mirrored in WAT of low n-6/n-3 diet fed individuals (Table 3). Interestingly, LA proportions in WAT increased by 14% on average, during the hibernation experiments, across all three dietary groups, although LA proportions only significantly differed in dormice fed an intermediate n-6/n-3 diet (Tables 3, 4). At mid-hibernation, the differences in the sum of n-6 or n-3 PUFA proportions as well as the n-6/n-3 ratio remained significant between the three dietary groups, while the sum of n-6 PUFAs and the n-6/n-3 ratio were increased by respectively 35% and 50% in WAT of dormice fed an intermediate or a high n-6/n-3 diet (Table 4). Further, proportions of 20:4 n-6 and 18:1 n-9 remained significantly lower and higher, respectively, in WAT of dormice fed a low n-6/n-3 diet than when fed a high n-6/n-3 diet, while WAT proportions of 20:4 n-6 were substantially reduced by 32% during hibernation in individuals fed a high n-6/n-3 lipid diet. Among SFAs, WAT proportions of several fatty acids were significantly affected, including 16:0 that decreased by 22% on average across all dietary groups, leading to 12% lowering of WAT proportions of the sum of SFA during the hibernation trial (Tables 3, 4). Also, dormice fed a low or an intermediate n-6/n-3 diet substantially reduced along hibernation their WAT proportions of long-chain fatty acids, i.e., 20:5 n-3, 22:5 n-3, and 22:6 n-3; levels which did not differ anymore significantly between the three dietary groups at mid-hibernation.

### Hibernating Patterns, Metabolic Rates, and Body Mass Loss

We found no significant differences of hibernating patterns during the experiments between dormice fed diets contrasting in their n-6/n-3 PUFA ratio during the fattening period (Table 5). Further, neither the mean torpid MR, nor the mean euthermic MR or hibernating MR, accounted for body mass variations, differed between the animals during the hibernation trial (Table 5). Although we observed a significant body mass loss in the hibernating dormice (pre- vs. mid-hibernation: 140.0 ± 2.6 g vs. 113.1 ± 2.3 g; F = 112.5, *p* < 0.001), the dietary treatment prior to hibernation did not affect body mass loss of the individuals over hibernation (Table 5).

**TABLE 5:** Variables of hibernating patterns, metabolic rate (MR), and overwinter body mass (means ± standard error) of garden dormice fed diets of either low (‘LOW’), intermediate (‘INTER’), or high n-6/n-3 PUFA ratio (‘HIGH’).

Variables	LOW	INTER	HIGH	ANOVA	
				*F*-value	*p*-Value
Hibernation Onset (days)	1.5 ± 0.3	1.6 ± 0.2	1.5 ± 0.1	0.55	0.58
Number of arousals	10.8 ± 0.7	10.3 ± 0.7	11.6 ± 0.7	1.3	0.28
Mean arousal duration (h)	6.9 ± 0.3	7.0 ± 0.3	6.4 ± 0.2	1.74	0.19
Total arousal duration (h)	73.0 ± 5.3	71.1 ± 5.4	74.0 ± 6.2	0.01	0.90
Mean torpor duration (h)	192.6 ± 6.8	193.7 ± 6.4	183.1 ± 10.4	0.50	0.61
Total torpor duration (h)	2171.2 ± 169.7	2117.2 ± 163.3	2241.4 ± 185.6	0.01	0.79
Minimal body temperature (°C)	4.31 ± 0.58	4.83 ± 0.21	4.02 ± 0.11	1.87	0.20
Mean torpid MR (ml O_2_ h^–1^)	6.1 ± 0.5	5.3 ± 0.6	6.4 ± 0.6	0.98	0.39
Mean euthermic MR (ml O_2_ h^–1^)	321.0 ± 23.0	288.7 ± 23.7	319.6 ± 18.9	0.69	0.51
Mean hibernating MR (ml O_2_ h^–1^)	18.1 ± 1.5	14.7 ± 1.9	17.5 ± 2.0	1.27	0.30
Mid-hibernation body mass (g)	110.4 ± 3.6	115.6 ± 4.0	112.7 ± 4.5	2.14	0.14

*Pre-hibernation body mass was accounted as a random factor in models for hibernating patterns and as a fixed factor in the model for body mass at mid-hibernation. Body mass was also entered as random factor in models for MRs. Further, hibernation duration was included as a random factor in the model for number of arousals as well as for total arousal and torpor durations. Significant p-Values are shown in italic. Groups differing significantly with p < 0.05 (Tukey-like post hoc comparisons) are denoted by different superscript letters when it applies.*

### Expression and Activity Levels of PPARs, PGC-1 α, and L-FABP During Hibernation

**FIGURE 1:**
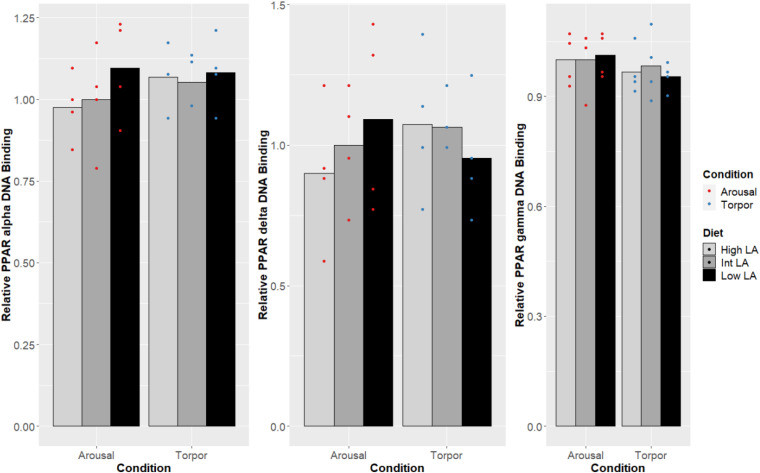
Response of PPAR pathway targets in GD WAT to low, intermediate or high levels of LA (or of mirrored levels of ALA) within the pre-hibernation diet. Histogram shows the relative DNA-binding levels, assessed as the average absorbance of the intermediate diet in the euthermic animals relative to the other conditions. Data are presented as the mean along with individual data points (*n* = 4), where a significant difference is shown by a difference in the label above the corresponding bar, as assessed by one-way ANOVA and Tukey’s *post hoc* analysis (*P* < 0.05). The absence of a label indicates no significant difference.

**FIGURE 2:**
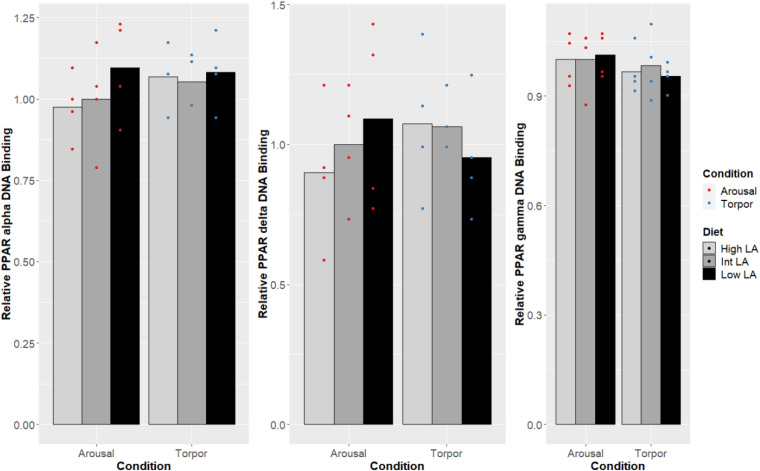
Response of PPAR pathway targets in GD BAT to low, intermediate or high levels of LA (or of mirrored levels of ALA) within the pre-hibernation diet. Histogram shows the relative DNA-binding levels, assessed as the average absorbance of the intermediate diet in the euthermic animals relative to the other conditions. Data are presented as the mean along with individual data points (*n* = 4), where a significant difference is shown by a difference in the label above the corresponding bar, as assessed by one-way ANOVA and Tukey’s *post hoc* analysis (*P* < 0.05). The absence of a label indicates no significant difference.

**FIGURE 3:**
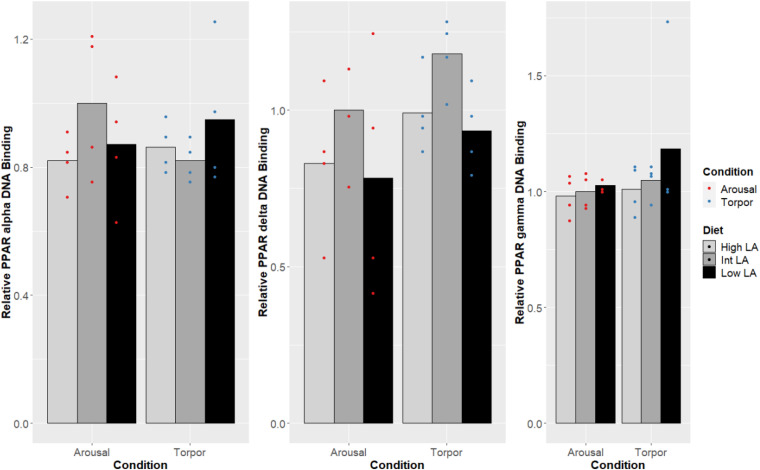
Response of PPAR pathway targets in GD liver to low, intermediate or high levels of LA (or of mirrored levels of ALA) within the pre-hibernation diet Histogram shows the relative DNA-binding levels, assessed as the average absorbance of the intermediate diet in the euthermic animals relative to the other conditions. Data are presented as the mean along with individual data points (*n* = 4), where a significant difference is shown by a difference in the label above the corresponding bar, as assessed by one-way ANOVA and Tukey’s *post hoc* analysis (*P* < 0.05). The absence of a label indicates no significant difference.

Notably, no changes were seen in the DNA-binding activity of PPARα, PPARδ, PPARγ due to the GD’s given diet and this was true during both the euthermic and torpid conditions in WAT (Figure 1), BAT (Figure 2), and liver (Figure 3). Similarly, comparing euthermic and torpid animals that were given the same diet condition, PPAR DNA-binding activity did not change.

**FIGURE 4:**
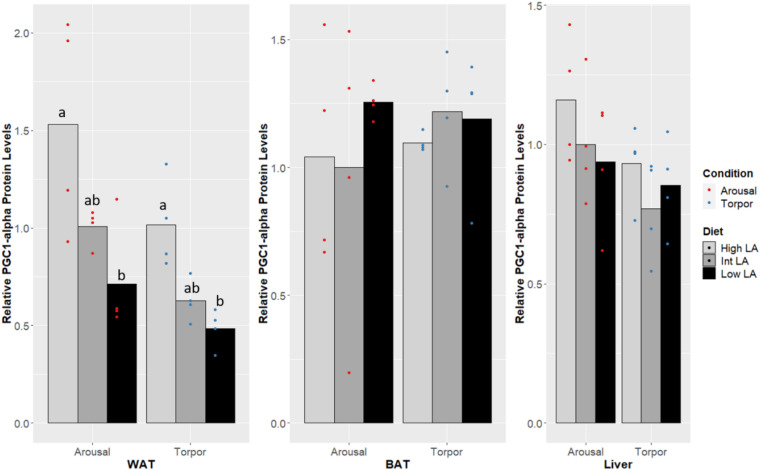
Response of PGC-1α in GD to low, intermediate or high levels of LA (or of mirrored levels of ALA) within the pre-hibernation diet. Histogram shows the relative protein amount, assessed as the average absorbance of the intermediate diet in the euthermic animals relative to the other conditions. Data are presented as the mean along with individual data points (*n* = 4), where a significant difference is shown by a difference in the label above the corresponding bar, as assessed by one-way ANOVA and Tukey’s *post hoc* analysis (*P* < 0.05). The absence of a label indicates no significant difference.

The co-transcription factor PGC-1α was found to differ in WAT (Figure 4). The amount of protein expressed in the low LA (or high ALA) condition was significantly reduced from levels expressed when the dormouse is fed a diet enriched with additional LA (low ALA or high n-6/n-3). A significant increase in PGC-1 protein amount was observed in both euthermic and torpid GD fed a high LA (or low ALA) diet compared to GD fed a low LA (or high ALA) diet; however, PGC-1α protein levels in either of the enriched diets were not significantly different from the intermediate LA (or high ALA) diet condition. Additionally, this WAT-specific difference was visible in both euthermic and torpid animals. PGC-1α levels were not different between the aroused and torpid state in WAT, and so the diet-induced changes were seen in both states.

**FIGURE 5:**
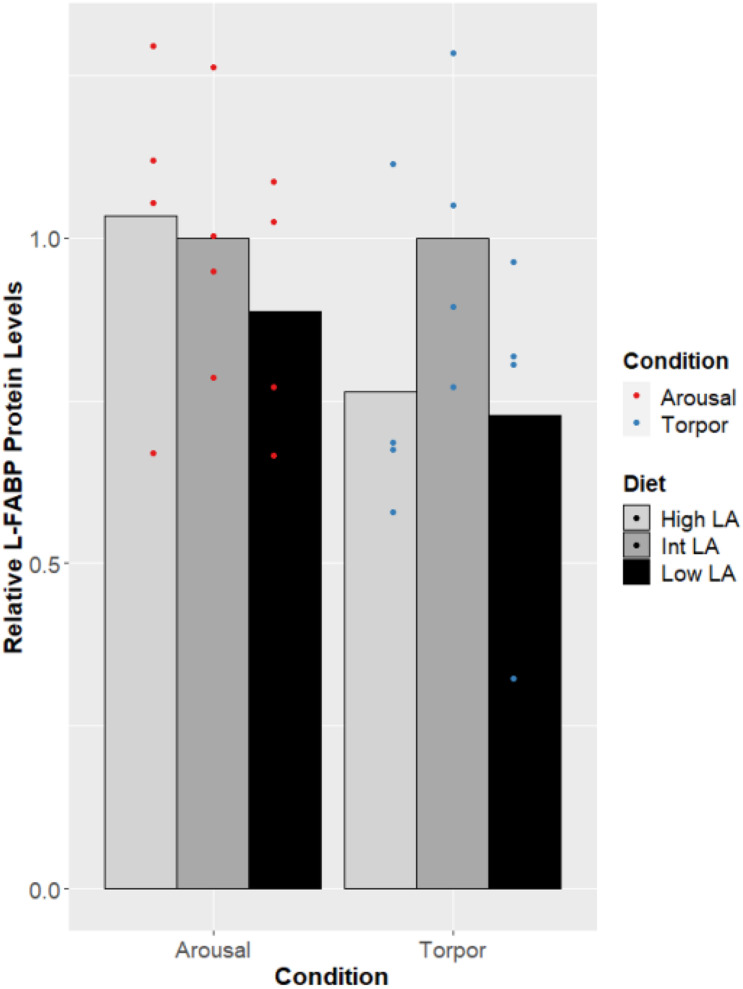
Response of Liver FABP in GD to low, intermediate or high levels of LA within the pre-hibernation diet. Histogram shows the relative protein amount, assessed as the average absorbance of the intermediate diet in the euthermic animals relative to the other conditions. Data are presented as the mean along with individual data points (*n* = 4), where a significant difference is shown by a difference in the label above the corresponding bar, as assessed by one-way ANOVA and Tukey’s *post hoc* analysis (*P* < 0.05). The absence of a label indicates no significant difference.

Finally, L-FABP displayed no changes across the different n-6/n-3 ratio diets (Figure 5), nor was a difference in the expression of FABP seen when comparing euthermic to torpid liver within a single diet condition.

## Discussion

In both torpid and euthermic GD, PGC-1α protein levels increased significantly within WAT in animals fed a high LA (or low ALA) diet compared to the animals fed a reduced amount of LA or increased level of ALA, while the intermediate group showed protein levels in between both groups (Figure 4). Interestingly, the pattern seen in torpid dormice was almost identical to that seen in euthermic animals, albeit at a slightly reduced signal level. Differences in PGC-1α protein levels induced by a high LA (or low ALA) diet suggest that induction of PPAR downstream targets is possible even though a difference in PPAR protein levels is not seen (Puigserver et al., 1998; for review, see Liang and Ward, 2006). As a co-activator of PPARγ target genes, PGC-1α is a central regulator of lipid-based energetics by stimulating mitochondrial metabolism in adipose tissue, and has been shown to play a role in the stimulation of a brown fat-like phenotype, or “beiging” within WAT (Puigserver and Spiegelman, 2003; Bargut et al., 2017), which is particularly interesting in the context of hibernation and warrants further study. For instance, increased levels of PGC-1α during hibernation in garden dormouse fed a high LA (or low ALA) diet could lead to co-activation of either PPARα or PPARγ, downstream activation of the mitochondrial transcription factor A (TFAM) and increased transcription of uncoupling protein-1 (UCP1); ultimately these changes would confer a more robust thermogenic capacity in animals fed a high LA (or low ALA) diet. This result is in line with finding from Logan et al. (2020) reporting increased levels of anti-oxidative and anti-apoptotic factors in the same dormice fed a high LA (or low ALA) diet compared to low or intermediate LA (or high or intermediate ALA) dietary levels during hibernation (euthermia). Further, PGC-1α in cooperation with PPARα, can increase PRD1-BF1-RIZ1 homologous domain-containing 16 (PRDM16) transcription, essential for the development and maintenance of “beige”-adipose tissue within WAT (Hondares et al., 2011; Ohno et al., 2012). PGC-1α levels are, therefore, deeply connected to the pre-hibernating diet choices in GD and its expression is likely consequential for WAT. Finally, the expression of PGC-1α is downstream of activated activating transcription factor-2 (ATF-2), which itself is downstream of activation by p38 mitogen-activated protein kinase (p38 MAPK) (Robidoux et al., 2005). Notably, p38 MAPK activation has previously been implicated in the maintenance of hibernating tissue (Eddy and Storey, 2007), and may potentially add to the protective effects elicited by PGC1α within hibernating tissues. Interestingly, UCP1 and electron transport chain protein levels are increased before the onset of hibernation within BAT, as shown in the 13-lined ground squirrel (Hindle and Martin, 2014), and remain heightened throughout the season, therefore hiding increases in the necessary upstream factors, such as PGC1α, from studies comparing protein levels between timepoints during hibernation.

The maintenance of PPAR DNA-binding activity across a range of LA or ALA levels within the diet during hibernation is intuitive given the dormouse’s requirement for lipid-based metabolism; differences in PPAR DNA-binding activity were not observed when comparing animals fed an intermediate LA or ALA diet to animals fed a diet enriched for either LA or ALA (Figures 2-4). Such observation is corroborated by the significant decrease of all WAT-proportions of SFA, including that of palmitic acid (16:0), as short-chain SFAs are preferentially mobilized for oxidation at lower energetic costs during hibernation (for review, see Dark, 2005). Similarly, total, PPAR protein levels were expected to differ with torpor and diet conditions since PPARα protein levels have been shown to increase during torpor (Han et al., 2015), levels of PPARγ are known to favor increased thermogenic mechanisms during torpor in hibernating species (Eddy and Storey, 2003; Kabine et al., 2004; Eddy et al., 2005), and studies within hibernating jerboa (*Jaculus orientalis*) show that a second truncated isoform, interferes with wild-type PPARα transcriptional activation during hibernation (Louet et al., 2001; El Kebbaj et al., 2009). Together, these results suggest that alternate mechanisms may be sufficient for prioritizing fatty acid catabolism during hibernation, without requiring changes to PPAR levels or DNA-binding activity. For instance, carbohydrate metabolism is inhibited via differential phosphorylation of metabolic enzymes and transcription factors in torpor (Buck et al., 2002; McMullen and Hallenbeck, 2010), forcing cellular metabolism to favor lipids as fuels. Similarly, differences in dietary preferences can partly explain the preferential catabolism of lipids as PPARδ-imposed suppression upon other PPARs is relieved by metabolites of LA catabolism, 13-S-hydroxyoctadecadienoic acid (13-S-HODE) (Shureiqi et al., 2003; Zuo et al., 2006). More specifically, LA is metabolized into arachidonic acid (AA), and AA-derived eicosanoids such as 13-S-HODE reduce PPARδ inhibition of other PPARs (Zuo et al., 2006). Furthermore, other AA-derived eicosanoids such as 8(S)-hydroxyeicosatetraenoic acid and Leukotriene B4 are more potent PPARα activators than n-3 lipids (Schmitz and Ecker, 2008). Therefore, our specific finding that LA 18:2 n-6 proportions increased significantly in dormice WAT during hibernation suggests that LA metabolites could regulate PPAR signaling pathways. Finally, the differential affinity of some PPAR co-activators for n-3 lipid-ligands over n-6 lipids (Schmitz and Ecker, 2008) could also affect PPAR target gene selection and is worth investigation in this model.

Finally, protein amounts of L-FABP were not changed by the GD’s dietary lipid composition or by hibernation. Given the results from liver which show a lack of changes in PPAR protein levels and PGC levels, the consistency of L-FABP protein levels is unsurprising, since L-FABP is downstream of PPARα and PPARδ-activated transcription (Ramiah et al., 2015). Remarkably, research has shown that isolated L-FABP from ground squirrel has an increased capacity for binding fatty acids across the range of temperatures experienced during hibernation compared to L-FABP isolated from rat and this difference accommodates an increased palmitate (16:0) binding capacity at torpid temperatures compared to euthermic temperatures (Stewart et al., 1998). Whether L-FABP from GD shares similar modifications is yet to be explored although it is likely that these differences would be favored in a hibernating mammal given their dependence on lipid metabolism. Furthermore, rat L-FABP does not have a higher affinity for n-3 PUFAs over n-6 PUFAs but dormouse L-FABP function could be regulated by differential levels of fatty acid species or increases in absolute levels of fatty acids required for torpor (Norris and Spector, 2002).

The presence of n-3 fatty acids can adversely affect a mammal’s hibernation and this has been demonstrated to be true within a number of hibernating species (Frank et al., 2004; Ruf and Arnold, 2008; Diedrich et al., 2014). Differences in the ratio of n-6 to n-3 fatty acids within hibernators’ diets modulates the deposition of adipose within depots as well as the total amount of adipose tissue within the animal (Frank et al., 1998), that can lead to downstream effects on a hibernator’s ability to adapt to winter. Marmots that ingest a higher amount of ALA relative to LA spend less time in hibernation, incorporate a higher amount of n-3 PUFAs into their WAT, and ingest more food in winter (Hill and Florant, 2000). This is in contrast with our observations in this study during which dormice hibernated in the same way without food supply and regardless of the dietary lipid manipulation applied prior to hibernation. Dormice of the present study were hibernating at T_*a*_ of 4°C, with a T_*b*_ of ∼0.5°C above ambient (barely above T_*a*_ for dormice fed a high LA diet). Then, animals were mostly thermoconforming during hibernation, and such thermal conditions might have been associated with rather small (not detectable) differences between dietary groups. Interestingly, such differences can become visible and significant when torpid individuals are exposed to low Ta (Geiser et al., 1997) when animals need to thermoregulate (Barnes and Buck, 2000; Buck and Barnes, 2000). In such case, the effects of dietary intake prior to hibernation might constrain hibernation performances with important ecological implications, including accelerated use of energetic fuels and a more rapid depletion of energy (fat) reserves, which can impair individual’s survival during winter hibernation. Nevertheless, no such differences in thermal and metabolic patterns were reported under the hibernating conditions of the dormice fed contrasted lipid diets during pre-hibernation in this study. Instead, animals seemed to have specifically remodeled their WAT lipid composition reducing levels of all n-3 PUFA, including long-chain fatty acids, along with enrichment of WAT-LA during hibernation. Such process of lipid remodeling independent of diets during hibernation has previously been observed in hibernating alpine marmots (Arnold et al., 2011) as well as in hibernating brown bears (Giroud et al., 2019). Nevertheless, feeding low n-6/n-3 diets (high ALA content) to dormice affected neither the torpor patterns nor the onset of hibernation of individuals in this study. This is again in contrast with our previous study where dormice that ingested a higher amount of docosahexaenoic acid (DHA, 22:3 n-3), relative to LA, during the fattening phase significantly delayed the onset of hibernation and had a higher proportion of n-3 fatty acid within their WAT and cardiac sarcoplasmic reticulum (SR) membranes (Giroud et al., 2018). This is important as low levels of n-6 PUFAs and/or high amounts of n-3 PUFAs can alter calcium reuptake within the heart (Swanson et al., 1989; Taffet et al., 1993), and prolong entrance into torpor due to inefficient cardiac maintenance and functioning (Hill and Florant, 2000; Ruf and Arnold, 2008). Such difference of an effect on hibernating patterns observed between our previous study (Giroud et al., 2018) and the present investigation would likely be explained by differential actions of DHA 22:6 n-3 and ALA 18:3 n-3 on oxidative metabolic pathways during hibernation. Clearly, further research is needed on how dietary lipids can affect hibernation performances of species, including modulations of both hibernating patterns and specific use of lipids or other energetic substrates.

In summary, PPAR transcriptional activation seems to be remarkably resilient to differences in the dietary n-6/n-3 ratio and to torpor. The overall molecular phenotype observed suggests that DNA binding activity is maintained within the family of PPAR transcription factors, and at levels similar to that seen in the euthermic GD. It is possible that the GD begins to increase PPAR activities and display a profile favoring lipid catabolism, possibly with preferential utilization of certain lipid types, across the entire hibernation season. If this were the case DNA-binding activity would likely show significant differences when comparing a euthermic GD during the active season, as seen in other hibernating species (Kabine et al., 2004; Chayama et al., 2018) although this comparison is out of the scope of the present study. Future studies should also address potential differences in peroxisomal lipid-metabolism in the hibernating GD given the mitochondria is less reliant on PPAR transcriptional activation, which was maintained across diets and torpor.

## Data Availability Statement

The original contributions presented in the study are included in the article/supplementary materials, further inquiries can be directed to the corresponding author/s.

## Ethics Statement

The animal study was reviewed and approved by the institutional ethics committee and the national Austrian authority in accordance to the Austrian Animal Experimentation Act, “Tierversuchsgesetz 2012” (BMBWF-68.205/0137-WF/V/3b/2014).

## Author Contributions

AW, SL, SG, and KS did the conceptualization. AP, AK-H, and SG performed the methodology (model creation, animal setup, and experiments). JP and GS performed the surgeries. AW, SL, and KS performed the methodology (molecular analyses). AW, SL, and SG investigated the data. AW and SL validated the data. AW, KG, and SG carried out the formal analysis. AW visualized the data. SG and KS carried out the resources and funding acquisition and supervised the data. AW and SG prepared the manuscript. AW, SL, KG, SG, and KS reviewed and edited the manuscript. KS carried out the project administration. All authors commented and agreed on the final version of the manuscript and participated in revisions.

## Conflict of Interest

The authors declare that the research was conducted in the absence of any commercial or financial relationships that could be construed as a potential conflict of interest.

## Footnotes

1 https://www.sablesys.com
